# Evolution of the avian digital pattern

**DOI:** 10.1038/s41598-019-44913-w

**Published:** 2019-06-12

**Authors:** Kenta Kawahata, Ingrid Rosenburg Cordeiro, Shogo Ueda, Guojun Sheng, Yuuta Moriyama, Chika Nishimori, Reiko Yu, Makoto Koizumi, Masataka Okabe, Mikiko Tanaka

**Affiliations:** 1 School of Life Science and Technology, Tokyo Institute of Technology, Yokohama, Japan; 2 Present Address: International Research Center for Medical Sciences, Kumamoto University, Kumamoto, Japan; 3 Laboratory Animal Facilities, The Jikei University School of Medicine, Tokyo, Japan; 4 Department of Anatomy, The Jikei University School of Medicine, Tokyo, Japan; 5 Present Address: Laboratory for Immunotherapy, RIKEN Center for Integrative Medical Sciences, Yokohama, Japan; 6 RIKEN Center for Developmental Biology, Kobe, Japan; 7 Present Address: Department of Physics and Mathematics, College of Science and Engineering, Aoyama Gakuin University, Sagamihara, Japan

**Keywords:** Pattern formation, Evolutionary developmental biology, Evolutionary developmental biology, Pattern formation

## Abstract

Variation in digit number has occurred multiple times in the history of archosaur evolution. The five digits of dinosaur limbs were reduced to three in bird forelimbs, and were further reduced in the vestigial forelimbs of the emu. Regulation of digit number has been investigated previously by examining genes involved in anterior-posterior patterning in forelimb buds among emu (*Dromaius novaehollandiae*), chicken (*Gallus gallus*) and zebra finch (*Taeniopygia guttata*). It was described that the expression of posterior genes are conserved among these three birds, whereas expression of anterior genes *Gli3* and *Alx4* varied significantly. Here we re-examined the expression pattern of *Gli3* and *Alx4* in the forelimb of emu, chicken and zebra finch. We found that *Gli3* is expressed in the anterior region, although its range varied among species, and that the expression pattern of *Alx4* in forelimb buds is broadly conserved in a stage-specific manner. We also found that the dynamic expression pattern of the BMP antagonist *Gremlin1* (*Grem1*) in limb buds, which is critical for autopodial expansion, was consistent with the digital pattern of emu, chicken and zebra finch. Furthermore, in emu, variation among individuals was observed in the width of *Grem1* expression in forelimb buds, as well as in the adult skeletal pattern. Our results support the view that the signalling system that regulates the dynamic expression of *Grem1* in the limb bud contributes substantially to variations in avian digital patterns.

## Introduction

Variation in digit number has been observed several times in the lineage of the Archosauria. The number of digits in the forelimb was reduced from five to three during the transition from dinosaurs to birds, and it was further reduced in the flightless emu^1^. Numerous studies have explored the evolutionary history of the alteration of digit numbers seen in avian lineages^2–8^, whose developmental basis are still under debate.

In vertebrates, limb development is orchestrated by a self-regulatory signalling system operated by feedback loops, the SHH/GREM1/AER-FGF system^9^. It is comprised by two major signalling centres—the SHH-producing zone of polarizing activity (ZPA) in the posterior mesenchyme and the FGF- producing apical ectodermal ridge (AER). Gremlin1 (Grem1) is the key node of this feedback loop, acts as a relay signal between these two signalling modules and positively regulating proliferation of limb bud cells by inhibition of BMP signalling^10–12^. With the expansion of the limb, the Grem1 expression terminates, leading to increased BMP signalling and chondrogenic differentiation of the digital primordia^13^. Thus, the SHH/GREM1/AER-FGF system is critical to regulate the number of digits by regulating the amount of digit progenitors, which is indicated by the GREM1 expression during the limb outgrowth phase^10,14–17^.

Previous work by de Bakker *et al*.^8^ described the expression patterns of the posterior genes *Hoxd11* and *Hoxd12* as being conserved in forelimb buds among emu (*Dromaius novaehollandiae*), chicken (*Gallus gallus*) and zebra finch (*Taeniopygia guttata*), whereas expression of anterior genes *Gli3* and *Alx4* varied significantly. Those authors concluded that the rapid loss of the anterior digit may reflect weaker developmental constraints, while the specification of the posterior digits is ZPA-dependent and thus more constrained^8^. Here we re-examined the expression patterns of the anterior genes *Gli3* and *Alx4* in limb buds of emu, chicken and zebra finch embryos. Our results suggest that the forelimb buds of emu used for the previous work^8^ were from embryos older than the other two species investigated, and thus the expression patterns of emu *Gli3* and *Alx4* differ from those described previously. In particular, the expression of *Alx4* in forelimb buds is broadly conserved across species in a stage-sensitive manner. We also found that the dynamic expression pattern of *Grem1* in early limb buds is consistent with the avian digital patterns. These results support the view that the signalling system regulating dynamic expression of *Grem1* in the limb bud contributes substantially to variations in the digital patterns among avian species.

## Results and Discussion

**Figure 1:**
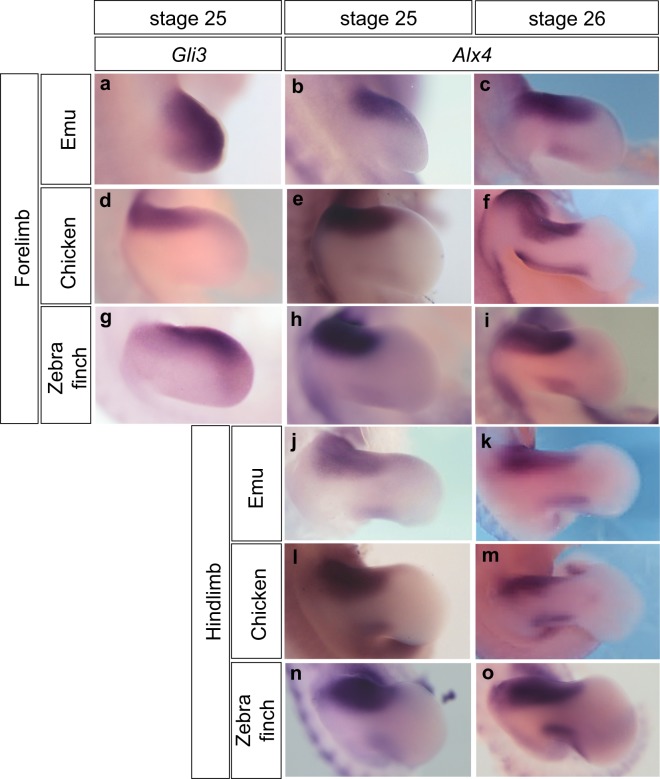
Expression patterns of *Gli3* and *Alx4* in limb buds of emu, chicken and zebra finch embryos. The distal domain of *Gli3* expression is posteriorly extended in limb buds of emu, chicken and zebra finch (**a**, n = 5; **d**, n = 9; **g**, n = 8), although it is most extensively expressed in the emu forelimb buds. *Alx4* show a similar anterior expression in limb buds of all species (**b**, **j**, n = 5; **e**, **l**, n = 17; **h**, **n**, n = 3) at stage 25. Additional posterior expression of *Alx4* is detected in both fore- and hindlimb buds of emu, chicken and zebra finch embryos at stage 26 (**c**, **k**, n = 6; **f**, **m**, n = 13; **i**, **o**, n = 7). The shapes of the limb bud are similar, but not exactly identical among species at the same stage^14,22,23^. **c**, **d**, **j**, **k**, Left limb buds flipped horizontally.

First, we re-examined the expression patterns of *Gli3* and *Alx4* in limb buds of emu, chicken and zebra finch embryos (Figs 1, S1, S2). To ensure an accurate staging of all embryos, the hindlimb shape was used as morphological criteria for identifying the Hamburger-Hamilton stages in chicken^18^, which was adapted for staging zebra finch^19^ and emu^20^ embryos. Specifically, stage 25 is defined by a faint demarcation of one digit in the hindlimb plate, and stage 26 by three digit indentations clearly visible in the hindlimbs.

*Gli3* expression was extensively expressed in the emu forelimb buds at stage 25, although it was more intense in the anterior region (Fig. 1a). In the chicken forelimb bud, *Gli3* expression was detected in the anterior region (Fig. 1d), while it was extended posteriorly in the forelimb bud of zebra finch (Fig. 1g). Although the stage of the emu embryos was different from that in the previous study^8^, our data also suggest that the extent of *Gli3* expression in limb buds vary among emu, chicken and zebra finch.

In contrast, the expression pattern of *Alx4* in the forelimb bud was broadly conserved among emu, chicken and zebra finch. We detected the transcripts of *Alx4* in the anterior one-third of the emu forelimb buds at stage 25 (Fig. 1b) as well as in chicken and zebra finch forelimb buds (Fig. 1e,h). The posterior expression of *Alx4* reported in the emu forelimb bud^8^ was also seen in both chicken and zebra finch at stage 26 (Fig. 1c,f,i), as well as in their hindlimb buds (Fig. 1j–o). Similar posterior expression of *Alx4* was previously shown in the chicken forelimb bud at late stage 25^21^. Although it was reported that *Alx4* expression extended posteriorly (similar to *Gli3* expression) in the zebra finch^8^, we detected *Alx4* transcripts in the anterior one-third of wing buds (Fig. 1h). Therefore, the anterior *Alx4* expression in forelimb buds was broadly conserved among emu, chicken and zebra finch in a stage-specific manner.

**Figure 2:**
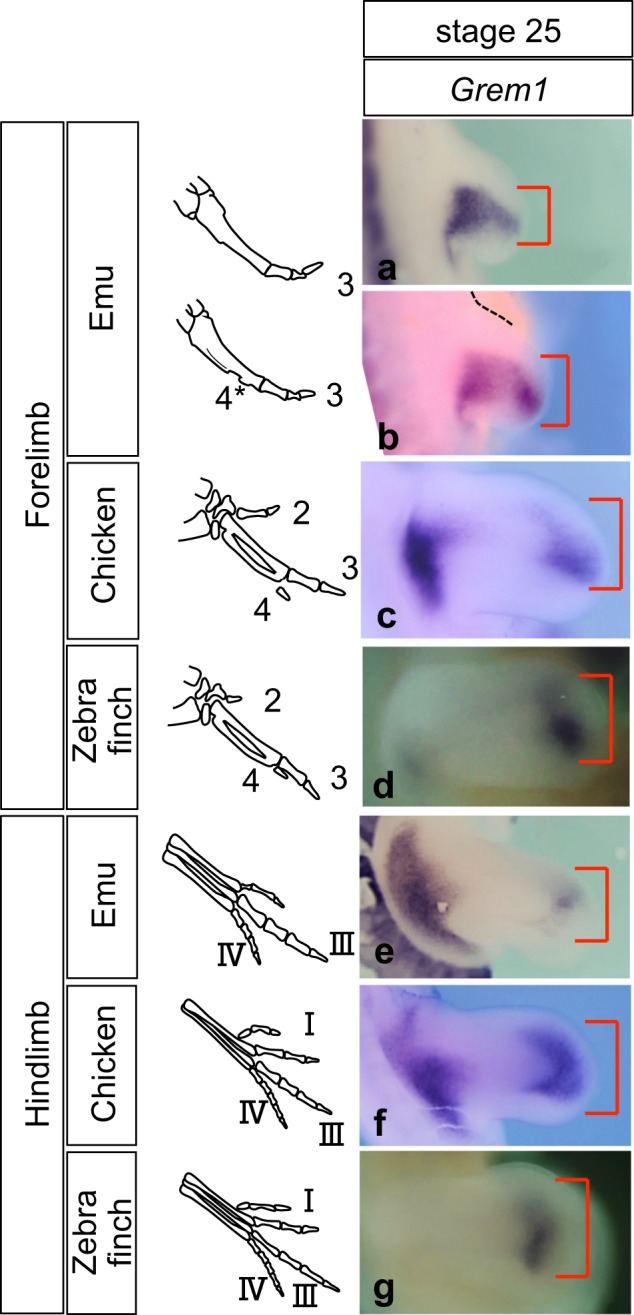
Expression patterns of *Grem1* in limb buds is consistent with the resulting skeletal pattern. (**a**−**g**) Distal *Grem1* expression in forelimb and hindlimb buds of stage 25 emu, chicken and zebra finch embryos is correlated with the digital skeletal patterns shown on the left (**a**, **b**, **e** n = 9; **c**, **f**, n = 10; **d**, **g**, n = 3). **a**, **b** Left limb buds flipped horizontally. Note that size of the distal *Grem1*-positive area (bracket) varies among emu embryos at the same stage.

**Figure 3:**
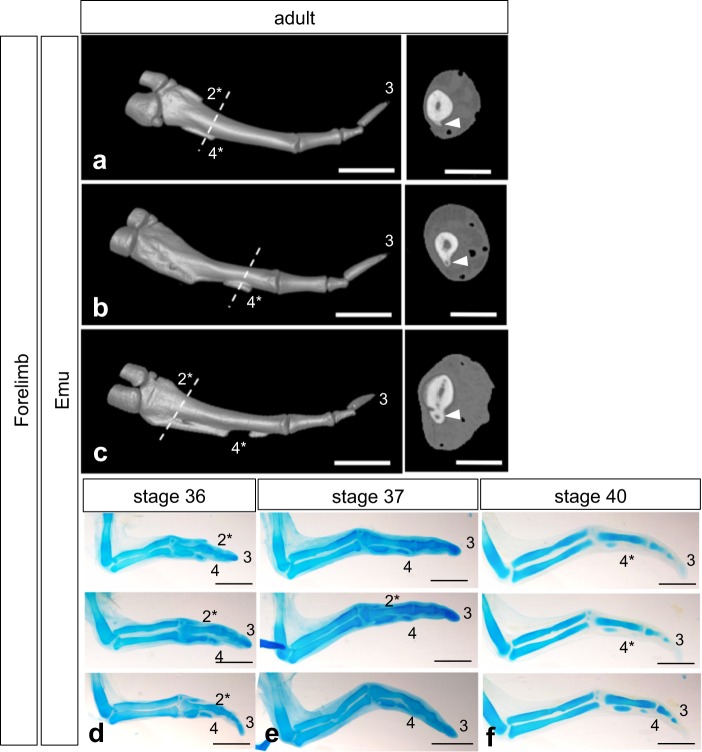
Digital pattern of the forelimb of emu adults and embryos. (**a–c**) Three-dimensional rendering from CT images of the digital plate of adult emu forelimb skeletons (left; ventral view) and their transverse sections taken from the limb at the level of the dashed line (right). Arrowheads indicate the vestigial digit 4 (4*). Medullar cavities are seen in the vestigial digit 4. (**c**) A right limb flipped horizontally. Scale bars, 20 mm (left) and 10 mm (right). (**d–f**) Alcian blue staining of wings of emu embryos at stages 36 (d), 37 (e) and 40 (f). Note that individual variation was recognised in the cartilage pattern of developing forelimbs (stage 36, n = 4; stage 37, n = 3; stage 40, n = 3). Left wings flipped horizontally. 2−4, digits 2−4; 2*, 4*, rudiment of digit 2 and 4, respectively. Scale bars, 2 mm.

We then aimed to understand the contribution of the SHH/GREM1/AER-FGF feedback loop^10,11^ to the variation in digital pattern among birds, focusing on the BMP antagonist Gremlin1, a key node of this system^10–12^. For this purpose, we isolated *Grem1* of each species (Fig. S3) and examined its expression pattern. The width of the *Grem1* expression domain in the limb bud was consistent with the resulting skeletal pattern (Fig. 2), supporting the view that the level of BMP activity is critical for creating variation in the digital pattern^14,17^. As Gli3, a key component of SHH signalling, directly controls the expression of *Grem1* in limb buds^22^, differences in the expression of *Gli3* in forelimb buds among emu, chicken and zebra finch observed here (Fig. 1a,d,g) are likely to contribute to the resulting distal *Grem1* expression patterns and digit number. Interestingly, in emu forelimb buds, the width of distal *Grem1* along the anterior-posterior axis varied among individuals (Fig. 2a,b). Distal *Grem1* expression area relative to total forelimb area showed a greater variation in emu (0.778 ± 0.264, mean ± s.d., n = 7) than in chicken (0.762 ± 0.114, mean ± s.d., n = 10) (Fig. S4). The skeletal elements of adult emu wings demonstrated a great range of individual variation as well (Fig. 3a–c)^23^. Among the 24 wings that we examined, 10 had a small rudiment of digit 2 fused to digit 3 in the proximal region, and 15 had a partial digit 4 at the posterior margin (Fig. 3a–c; digits are referred to by their embryological origin, not by their osteological identity). A high degree of individual variation was also recognised in the cartilage pattern of developing forelimbs (Fig. 3d–f), suggesting that this range of individual variation might already be present in early limb bud stages as indicated by *Grem1* expression. Our results suggest that the expression pattern of *Grem1* in the early limb bud reflects both intraspecific and interspecific variation in digital patterns in avian species.

In conclusion, our results support the hypothesis that variation in digit number arise from regulation of the feedback loops that promote limb outgrowth and patterning, the SHH/GREM1/AER-FGF system. The extent of the expression of *Gli3*, a key component of the SHH pathway, varied between emu, chicken and zebra finch (Fig. 1a,d,g). We agree with the interpretation that the spatiotemporal expression pattern of the anterior gene *Gli3* is critical for creating the variation in the resulting digital pattern, as it directly controls *Grem1* expression and digit number^10,17^.

Unlike previously reported^8^, the expression pattern of another anterior gene, *Alx4*, was broadly conserved across species^8^. A posterior expression domain of *Alx4* appears at stage 26 in the forelimb bud of all three birds, suggesting that it is not unique to emu. In addition, the emu forelimb bud has been proposed to develop heterochronically based on delayed SHH expression during the initiation of limb outgrowth^24^. The conserved expression pattern of *Alx4* observed in this study is inconsistent with this model at least at stages 25 and 26. Still, we do not exclude the possibility that slight differences in *Alx4* expression levels could affect the skeletal pattern. In fact, *Grem1* expression is upregulated in the anterior part of *Alx4* mutant limb buds^25^. It is also important to point out that anterior propagation of *Grem1* expression depends on the level of *HoxA* and *HoxD* expression^26^, and anterior skeletal elements can be influenced by subtle changes in the duration or level of posterior *Shh* expression^27^, even though de Bakker *et al*. suggested that *Hoxd11*, *Hoxd12* and *Shh* had similar expression patterns in emu, chicken and zebra finch^8^. Thus, differences in the expression of both anterior and posterior genes, which affects the expression of *Grem1*, can lead to the variation in the final digital pattern.

In this study, we showed that the spatiotemporal expression pattern of *Grem1* was highly consistent with the final digital pattern of birds. Furthermore, *Grem1* expression in the emu forelimb bud was consistent with their variation of the adult skeletal pattern. Experimental manipulations of *Grem1* expression alter the skeletal pattern in several models. The formation of additional phalanges can be induced by infection of chicken forelimbs with *Grem1*-expressing virus^14^. Furthermore, in mouse embryos, inhibiting BMP signalling throughout limb buds by overexpressing *Grem1* between E10.5 and E11.5 leads to the elongation of digits as well as the formation of both pre- and post-axial polydactylys^28^. In the limb buds, proliferation of mesenchymal cells terminates after the downregulation of *Grem1*, and subsequently these cells undergo chondrogenic differentiation^17,29^. It is more likely that the avian digital pattern is regulated by the well-documented SHH/Gremlin1/AER-FGF feedback loops, in which Gremlin1 is the critical node linking each signalling module^10,14–17^, and both intraspecific and interspecific variation in the digital pattern can be recognized as the expression pattern of *Grem1* in early limb buds.

Finally, a recent study showed that the co-option of *Nkx2*.*5* in the emu forelimb bud leads to the reduction of forelimb growth and digit loss; however, it remains unknown how Nkx2.5 inhibits the expansion of limb bud^30^. Future studies should determine whether *Nkx2*.*5* expression in emu forelimb bud leads to their extreme digit reduction via regulation of the SHH/GREM1/AER-FGF feedback loops, or via another pathway.

## Methods

### Data reporting

No statistical methods were used to predetermine sample size. The experiments were not randomised and the investigators were not blinded to allocation during experiments and outcome assessment. The sex of the embryos is unknown.

### Animals

Chicken (*Gallus gallus*) eggs were incubated at 38 °C and staged^18^. Fertilised emu (*Dromaius novaehollandiae*) eggs were purchased from Kakegawa Kachoen and Okhotsk Emu Pasture, incubated at 36.5 °C and staged as described^20^. Zebra finch (*Taeniopygia guttata*) eggs were collected, incubated at 38 °C and staged as described^19^. For *in situ* hybridization, embryos were fixed overnight in 4% paraformaldehyde in phosphate-buffered saline, dehydrated in a graded methanol series and stored in 100% methanol at −20 °C. All animal work was performed in accordance with guidelines for animal experiments of the Tokyo Institute of Technology, RIKEN, and The Jikei University School of Medicine, and the experimental protocols were approved by the committees of Tokyo Institute of Technology, RIKEN, and the Jikei University of Medicine.

### Gene isolation and phylogenetic analysis

Total RNA was extracted from stage 20 chick, stage 25 emu and stage 17−25 zebra finch embryos using RNeasy kit (Qiagen). cDNA was synthesised by reverse transcription and used as a template for PCR. To isolate emu, chick and zebra finch genes, we used the following avian universal primers: avian *Alx4*, 5′-CTACTACAACGCAGCCTCCC-3′ and 5′-CTTYGCTTTCATCCTCAGGGC-3′; avian *Gli3*, 5′- ATATCGCACCTTCCCGAACC-3′ and 5′-GATGAGTGGAGGGCTGTGTC-3′; avian

*Grem1*, 5′-TCCTGTCAAGGATCAGCCCA-3′ and 5′-ACACCGGCACTCCTTAACTC-3′. The gene fragments were cloned into pGEM T-easy vector (Promega). The partial coding sequences for *D*. *novaehollandiae Grem1*, *D*. *novaehollandiae Gli3*, *D*. *novaehollandiae Alx4*, *T*. *guttata Grem1*, *T*. *guttata Gli3*, and *T*. *guttata Alx4* were submitted to GenBank under accession numbers MH352496–MH352501, respectively. Amino acid sequences were aligned using ClustalW version 2.1^31^.

### Probe synthesis and *in situ* hybridisation

*D*. *novaehollandiae*, *G*. *gallus* and *T*. *guttata Alx4*, *Gli3* and *Grem1*, all of which were in pGEM T-easy vector, were used as templates for riboprobe synthesis. Whole-mount *in situ* hybridisation was carried out as described^32^. We used both *G*. *gallus* and *T*. *guttata Grem1* probes for expression analysis of *Grem1* in *T*. *guttata* embryos as they produced the same results.

### Measurement of *Grem1* expression ratio

Measurements were made using ImageJ (https://imagej.net/Downloads). The expression area and limb area were delimited manually. Then, the ratio between the distal *Grem1* expression area and whole limb area was calculated. To normalize any experimental variation during *in situ* hybridization staining, we divided the forelimb *Grem1* expression ratio by the hindlimb *Grem1* expression ratio of the same embryo. The *Grem1* expression ratio was defined as mean ± s.d.

### Alcian blue staining

Embryos were fixed in 4% PFA, stained in 0.1% Alcian blue in 1% HCl/70% ethanol, dehydrated in ethanol and cleared in methyl salicylate.

### Computed Tomography imaging

Computed Tomography (CT) Imaging was performed by Micro-CT system (Latheta LCT-200, Hitachi Aloka Medical Ltd., Tokyo, Japan) for adult emu forelimb skeletons. Acquired slice data were rendered as three-dimensional images using the VGStudio MAX2.0 software (Volume Graphics GmbH., Heidelberg, Germany).

## Supplementary information

Dataset1

## Data Availability

The authors declare that all data supporting the findings of this study are available within the article and its Supplementary Information Files or from the corresponding author upon reasonable request.
