# Supplementary material for: Evolution of the avian digital pattern
Source: Sci Rep. 2019 Jun 12;9:8560. doi: 10.1038/s41598-019-44913-w (PMC6561939; doi:10.1038/s41598-019-44913-w)
Supplement: Supplementary file 1 — Dataset1 [file 41598_2019_44913_MOESM1_ESM.pdf]

Evolution of the avian digital pattern

Kenta Kawahata[1,*], Ingrid Rosenburg Cordeiro[1,*], Shogo Ueda[1,5], Guojun Sheng[2,6], Yuuta Moriyama[1,7], Chika Nishimori[1], Reiko Yu[1], Makoto Koizumi[3], Masataka Okabe[4] & Mikiko Tanaka[1,†]

[1]School of Life Science and Technology, Tokyo Institute of Technology, B-17, 4259 Nagatsuta-cho, Midori-ku, Yokohama, Japan

[2]International Research Center for Medical Sciences, Kumamoto University, Kumamoto, Japan

[3]Laboratory Animal Facilities, The Jikei University School of Medicine, Tokyo, Japan

[4]Department of Anatomy, The Jikei University School of Medicine, Tokyo, Japan

[7]Current address: Laboratory for Immunotherapy, RIKEN Center for Integrative Medical Sciences (IMS), Yokohama, Japan

[6]Previous address: RIKEN Center for Developmental Biology, Kobe, Hyogo, Japan

[7]Current address: Department of Physics and Mathematics, College of Science and Engineering, Aoyama Gakuin University, Japan

*These authors contributed equally to this work.

[†]To whom correspondence should be addressed. *E-mail*: mitanaka@bio.titech.ac.jp

Supplementary figures

Figure S1 | Amino acid sequence alignment of the Gli3 proteins of emu, chicken, zebra finch and mouse.

Figure S2 | Amino acid sequence alignment of the Alx4 proteins of emu, chicken, zebra finch and mouse.

Figure S3 | Amino acid sequence alignment of the Gremlin1 proteins of emu, chicken, zebra finch and mouse.

Figure S4 | Variation in the width of the distal *Grem1* expression domain in chicken and emu wings.

```
emu-GLI3            ------------------------------------------------------------
chicken-GLI3        MEAQSHSSTTTEKKKVENSIVKCSNRTDVSEKAVASSTTSNEDESPGQTYHRERRNAITM 60
zebra finch-GLI3    ------------------------------------------------------------
mouse-GLI3          MEAQAHSSTATERKKAENSIGKCPTRTDVSEKAVASSTTSNEDESPGQIYHRERRNAITM 60

emu-GLI3            ---------------------------------ISHLPEPSVPYRGTLFTMDPRNGYMDP 27
chicken-GLI3        QPQGGQGLSKISEEPSTSSEERASLIKKEIHGSISHLPEPSVPYRGTLFTMDPRNGYMDP 120
zebra finch-GLI3    ---------------------------------ISHLPEPSVPYRGTLFTMDPRNGYMDP 27
mouse-GLI3          QPQSVQGLNKISEEPSTSSDERASLIKKEIHGSLPHLAEPSLPYRGTVFAMDPRNGYMEP 120

emu-GLI3            HYHPPHLFLAFHPPVPIDARHHEGRYHYEPSPIPPLHVPSALSSSPTYSDLPFIRISPHR 87
chicken-GLI3        HYHPPHLFPAFHPPVPIDARHHEGRYHYEPSPIPPLHVPSALSSSPTYSDLPFIRISPHR 180
zebra finch-GLI3    HYHPPHLFPAFHPPVPIDARHHEGRYHYEPSPIPPLHVPSALSSSPTYSELPFLRISPHR 87
mouse-GLI3          HYHPPHLFPAFHPPVPIDARHHEGRYHYDPSPIPPLHVPSALSSSPTYPDLPFIRISPHR 180

emu-GLI3            NPAATSESPFSPPHPYINPYMDYIRSLHSSPSLSMISAARGLSPTDAPHAGVSPAEYYHQ 147
chicken-GLI3        NPAAASESPFSTPHPYINPYMDYIRSLHSSPSLSMISAARGLSPTDAPHAGVSPAEYYHQ 240
zebra finch-GLI3    NPAATSESPFSTPHPYINPYMDYIRSLHSSPSLSMISAARGLSPTDAPHAGVSPAEYYHQ 147
mouse-GLI3          NPTAASESPFSPPHPYINPYMDYIRSLHSSPSLSMISAARGLSPTDAPHAGVSPAEYYHQ 240

emu-GLI3            MALLAGQRSPYADIIPSAATAGAGALHMEYLHAMDSARFPSPRLSARPSRKRTLSISPLS 207
chicken-GLI3        MALLAGQRSPYADIIPSAATAGAGALHMEYLHAMDSTRFPSPRLSARPSRKRTLSISPLS 300
zebra finch-GLI3    MALLAGQRSPYADIIPSAATAGAGALHMEYLHAMDSARFPSPRLSARPSRKRTLSISPLS 207
mouse-GLI3          MALLTGQRSPYADILPSAATAGAGAIHMEYLHAMDSTRFPSPRLSARPSRKRTLSISPLS 300

emu-GLI3            DHSFDLQTMIRTSPNSLVTILNNSRSSSSASGSYGHLSASAISPALSFTYPPTPVSLQQM 267
chicken-GLI3        DHSFDLQTMIRTSPNSLVTILNNSRSSSSASGSYGHLSASAISPALSFTYPPTPVSLQQM 360
zebra finch-GLI3    DHSFDLQTMIRTSPNSLVTILNNSRSSSSASGSYGHLSASAISPALSFTYPPTPVSLQQM 267
mouse-GLI3          DHSFDLQTMIRTSPNSLVTILNNSRSSSSASGSYGHLSASAISPALSFTYPSAPVSLH-M 359

emu-GLI3            HQQIISRQQTLGSAFGHSPPLI-------------------------------------- 289
chicken-GLI3        HQQIISRQQTLGSAFGHSPPLIHPAPTFPTQRPIPGIPSVLNPVQVSSGPSESTQQNKPT 420
zebra finch-GLI3    HQQIISRQQTLGSAFGHSPPLI-------------------------------------- 289
mouse-GLI3          HQQILSRQQSLGSAFGHSPPLIHPAPTFPTQRPIPGIPTVLNPVQVSSGPSESSQ-SKPT 418

emu-GLI3            ------------------------------
chicken-GLI3        SESAVSSTGDPMHNKRSKIKPDEDLPSPGA 450
zebra finch-GLI3    ------------------------------
mouse-GLI3          SESAVSSTGDPMHNKRSKIKPDEDLPSPGS 448
```

Figure S1 | Amino acid sequence alignment of the Gli3 proteins of emu, chicken, zebra finch and mouse. The GenBank accession numbers of chicken and mouse GLI3 are as follows: *Gallus gallus*, NP_001258832.1 and *Mus musculus*, NP_032156.2. Boxes represent the location of avian universal primers used for PCR.

```
emu-ALX4           -------------------YYNTASQGTEGSSPFRAFQASDKFSPAFLANKGQGFGDSSA 41
chicken-ALX4       -MNADTCVSYCDPAAMDS-YYNAASQGADGSSPFRAFQASDKFGPAFLAAKGQSFGDSGA 58
zebra finch-ALX4   -------------------YYNAASQDTEGSSPFRAFQASDKFSPTFLASKGQGFGDSST 41
mouse-ALX4         MNAETCVSYCESPAAAMDAYYSPVSQSREGSSPFRGFPGGDKFGTTFLSAGAKGQGFGDA 60

emu-ALX4           KCRGRYSQQECQSLD---GNVQAPSSAAAPASFSKYPPPPQ-------HLYMQRGPCKTP 91
chicken-ALX4       KCRSRYSPQECPSLD---GSGQAPGPAAPPPAFSKYPQPQQQPP----HLYMQRGPCKTP 111
zebra finch-ALX4   KCRSRYSQQECQSLD---GSVQAPGSAGSPASFNKYPPQQQPP-----HLYMQRGPCKTP 93
mouse-ALX4         KSRARYGAGQQDLAAPLESSSGARGSFNKFQPQPPTPQPPPAPPAPPAHLYLQRGACKTP 120

emu-ALX4           PESNLKLQESSG-HNGALQVPCYGKESSLGEADLQSSSDPSGMDSSYLSVKEAGVKVPQD 150
chicken-ALX4       PESNLKLQESGG-HNGALQVSCYGKESSLGEADLQPNADPSGMDSSYLSVKEAGVKVPQD 170
zebra finch-ALX4   PDSNIKLQESSG-HNGALQVSCYGKESGLGEPDLQPSSDPSAMDSSYLSVKEAGVKVPQD 152
mouse-ALX4         PDGSLKLQEGSGGHNAALQVPCYAKESNLGEPELPPDSEPVGMDNSYLSVKETGAKGPQD 180

emu-ALX4           RASTDLPSPMDKADSESNKGKKRRNRTTFTSYQLEELEKVFQKTHYPDVYAREQLAMRTD 210
chicken-ALX4       RASTDLPSPMDKADSESNKGKKRRNRTTFTSYQLEELEKVFQKTHYPDVYAREQLAMRTD 230
zebra finch-ALX4   RASTDLPSPMDKADSESNKGKKRRNRTTFTSYQLEELEKVFQKTHYPDVYAREQLAMRTD 212
mouse-ALX4         RASAEIPSPLEKTDSESNKGKKRRNRTTFTSYQLEELEKVFQKTHYPDVYAREQLAMRTD 240

emu-ALX4           LTEARVQVWFQNRRAKWRTRERFGQMQQVRTHFSTAYELPLLTRAENYAQIQNPSWIGNN 270
chicken-ALX4       LTEARVQVWFQNRRAKWRKRERFGQMQQVRTHFSTAYELPLLTRAENYAQIQNPSWIGNN 290
zebra finch-ALX4   LTEARVQAWFQNRRAKWRKRERFGQMQQVRTHFSTAYELPLLTRAENYAQIQNPSWIGNN 272
mouse-ALX4         LTEARVQVWFQNRRAKWRKRERFGQMQQVRTHFSTAYELPLLTRAENYAQIQNPSWIGNN 300

emu-ALX4           GAASPVPACVVPCDTVPSCMSPHAHP--HAAGGVSDFLSVSGPGSHVGQTHMGSLFGTAG 328
chicken-ALX4       GAASPVPACVVPCEPVPSCMSPHAHP--HAAGGVSDFLGVSSTGGHVGQTHVGGLFGTAG 348
zebra finch-ALX4   GAASPVPACVVPCETVPSCMSPHGHP--HAAGGVSEFLGVSGPGSHVGQIHMGGLFGTAG 330
mouse-ALX4         GAASPVPACVVPCDPVPACMSPHAHPPGSGASSVSDFLSVSGAGSHVGQTHMGSLFGAAG 360

emu-ALX4           ISPGLNGYELNSEPDRKTSSIAALRMKAK---------- 357
chicken-ALX4       IGPSLNGYELNSEPDRKSSSIAALRMKAKEHSAAISWAT 387
zebra finch-ALX4   MSPGLNGYELNSEPDRKTSSIAALRMKAK---------- 359
mouse-ALX4         ISPGLNGYEMNGEPDRKTSSIAALRMKAKEHSAAISWAT 399
```

Figure S2 | Amino acid sequence alignment of the Alx4 proteins of emu, chicken, zebra finch and mouse. The GenBank accession numbers of chicken and mouse ALX4 are as follows: *Gallus gallus*, NP_989493.1 and *Mus musculus*, NP_031468.1. Boxes represent the location of avian universal primers used for PCR.

```
emu-GREM1            -------------------------------------PVKDQPNDSEQTQTQQQPGSRQR 23
chicken-GREM1        MVRTLYAIGAVFLLTGFLLPTAEGRKRNRGSQGAIPPPDKDQPNDSEQMQTQQQSGSRHR 60
zebra finch-GREM1    -------------------------------------PVKDQPNDSEQMQTQQQSGSRHR 23
mouse-GREM1          MNRTAYTVGALLLLLGTLLPTAEGKKKGSQG--AIPPPDKAQHNDSEQTQSPPQPGSRTR 58

emu-GREM1            ERG--KGTSMPAEEVLESSQEALHITERKYLKRDWCKTQPLKQTIHEEGCNSRTIINRFC 81
chicken-GREM1        ERG--KGTSMPAEEVLESSQEALHITERKYLKRDWCKTQPLKQTIHEEGCNSRTIINRFC 118
zebra finch-GREM1    ERG--KGTSMPAEEVLESSQEALHITERKYLKRDWCKTQPLKQTIHEEGCNSRTIINRFC 81
mouse-GREM1          GRGQGRGTAMPGEEVLESSQEALHVTERKYLKRDWCKTQPLKQTIHEEGCNSRTIINRFC 118

emu-GREM1            YGQCNSFYIPRHVRKEEGSFQSCSFCKPKKFTTMTVTLNCPELQPPRKKKRITRVKECRC 141
chicken-GREM1        YGQCNSFYIPRHVRKEEGSFQSCSFCKPKKFTTMTVTLNCPELQPPRKKKRITRVKECRC 178
zebra finch-GREM1    YGQCNSFYIPRHVRKEEGSFQSCSFCKPKKFTTMTVTLNCPELQPPRKKKRITRVKECRC 141
mouse-GREM1          YGQCNSFYIPRHIRKEEGSFQSCSFCKPKKFTTMMVTLNCPELQPPTKKKRVTRVKQCRC 178

emu-GREM1            ------
chicken-GREM1        ISIDLD 184
zebra finch-GREM1    ------
mouse-GREM1          ISIDLD 184
```

Figure S3 | Amino acid sequence alignment of the Gremlin1 proteins of emu, chicken, zebra finch and mouse. The GenBank accession numbers of chicken and mouse GREM1 are as follows: *Gallus gallus,* NP_990309.1 and *Mus musculus*, NP_035954.1. Boxes represent the location of avian universal primers used for PCR.

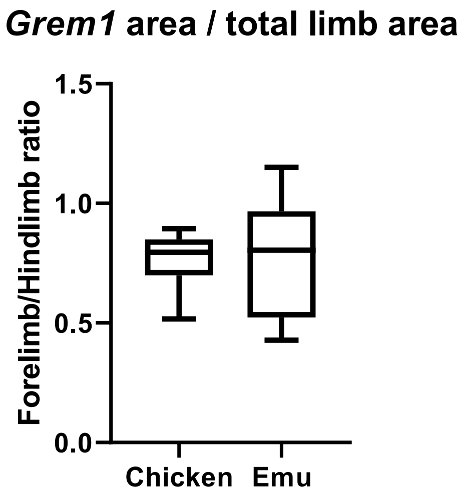

Figure S4 | Variation in the width of the distal *Grem1* expression domain in chicken and emu wings. The ratio between the distal *Grem1* expression area relative to total forelimb limb area had greater variation between emu embryos than chicken embryos. The forelimb ratio was divided by the hindlimb ratio in order to normalize possible experimental variations of the in situ hybridization staining.
